# COVID-19 susceptibility, severity, and vaccine effectiveness in patients with psoriasis: a nationwide cohort study in South Korea

**DOI:** 10.1038/s41598-025-06495-8

**Published:** 2025-07-15

**Authors:** Young Ah Cho, Hyein Han, Sungho Won, Ji Won Lim, Jae Young Sung, Chang Yong Kim, Da-Ae Yu, Yang Won Lee, Yong Beom Choe

**Affiliations:** 1 Department of Dermatology, Konkuk University School of Medicine, 120-1 Neungdong-ro, Gwangjin-gu, Seoul, 05030 Republic of Korea; 2 Department of Public Health Sciences, Seoul National University, 1 Gwanak-ro, Gwanak-gu, Seoul, 08826 Republic of Korea; 3 Institute of Health and Environment, Seoul National University, 1 Gwanak-ro, Gwanak-gu, Seoul, 08826 Republic of Korea; 4 RexSoft Inc, 542 Yeoksam-ro, Gangnam-gu, Seoul, 06187 Republic of Korea; 5 Research Institute of Medical Science, Konkuk University School of Medicine, 120-1 Neungdong-ro, Gwangjin-gu, Seoul, 05030 Republic of Korea

**Keywords:** Psoriasis, COVID-19, Susceptibility, Vaccine effectiveness, Immunosuppressive, Drugs, Biologics, Skin diseases, Viral infection

## Abstract

Patients with psoriasis are vulnerable to infections due to dysregulated immunity, use of immunosuppressive drugs, and comorbid conditions. Despite ongoing concerns regarding coronavirus disease 2019 (COVID-19) vulnerability in patients with psoriasis, evidence remains limited. This nationwide, population-based, retrospective cohort study aimed to assess the risk and severity of COVID-19 and vaccine effectiveness in patients with psoriasis, including those receiving immunomodulatory therapy, using Korean National Health Insurance Service claims data from 2018 to 2021. The primary analysis included 167,746 patients with psoriasis and 866,582 controls, revealing no significant associations between psoriasis and COVID-19 (adjusted hazard ratio [aHR]: 0.99, 95% confidence interval [CI]: 0.95–1.04). Conversely, the secondary analysis of 3,131 patients with psoriasis and 265,475 controls revealed significantly higher rates of severe COVID-19 in patients with psoriasis (aHR: 1.33, 95% CI: 1.08–1.64). While some treatment subgroups — such as the cyclosporine and methotrexate subgroups — demonstrated higher incidences of COVID-19 than the reference (nonsystemic) subgroup, these differences were not statistically significant. Furthermore, vaccine interaction effects between the duration of immunity and psoriasis or each treatment were insignificant (all *P* > 0.05). Patients with psoriasis exhibited a higher risk of severe COVID-19; however, their COVID-19 susceptibility and vaccine effectiveness resembled those of the control group. Additionally, the use of immunomodulatory agents did not impact COVID-19 risk or vaccine effectiveness. These findings highlight the need for dermatologists to implement pre- and postinfection strategies tailored for patients with psoriasis.

**Supplementary Information:**

The online version contains supplementary material available at 10.1038/s41598-025-06495-8.

## Introduction

Coronavirus disease 2019 (COVID-19), caused by severe acute respiratory syndrome coronavirus 2 (SARS-CoV-2), has affected approximately 800 million individuals worldwide. In South Korea, approximately 35 million people have been infected, resulting in 35,000 deaths as of July 2023 (World Health Organization COVID-19 Dashboard).

Psoriasis, a multisystem autoinflammatory disease, affects approximately 0.45% of the South Korean population^1^. Patients with psoriasis are vulnerable to infections due to dysregulated immunity, use of immunosuppressive drugs, and comorbid conditions. Notably, numerous studies have demonstrated an elevated risk of serious infections in this patient population^2–6^.

Despite concerns regarding the potential susceptibility of patients with psoriasis to SARS-CoV-2 infection, relevant data are limited. A previous review indicated no heightened vulnerability or severe outcomes of COVID-19 in patients with immune-mediated inflammatory diseases (IMIDs), including psoriasis^7^. Conversely, recent studies have reported an elevated risk of severe COVID-19 progression among patients with IMIDs^8,9^. However, most of these studies focused specifically on the risk of COVID-19 associated with psoriasis treatments, rather than assessing the overall risk of infection or disease severity in the broader psoriasis population.

This study aimed to investigate COVID-19 vulnerability and severity in patients with psoriasis, including those undergoing immunomodulatory therapy. We used data from the Korean National Health Insurance Service (NHIS) claims database to examine whether psoriasis and immunomodulatory therapies affect the effectiveness of COVID-19 vaccines.

## Materials and methods

### Data source

This study leveraged the database maintained by the Korea Disease Control and Prevention Agency (KDCA) and NHIS (Research No. KDCA-NHIS-2023-1-610). The NHIS in Korea provides coverage to 97% of the entire population and is responsible for collecting, processing, and summarizing all associated healthcare records. This dataset, primarily intended for academic research, comprises comprehensive demographics, diagnoses, prescriptions, and health examination data. The KDCA furnishes detailed COVID-19 information — including the route of infection, date of confirmation, and date of death — which is then integrated with the original data by the NHIS.

### Study population and settings

This retrospective, population-based cohort study included data for all adults with psoriasis who underwent health screening between 2018 and 2021. Psoriasis was defined based on the following International Classification of Diseases, 10th revision, codes (appearing at least twice prior to the index date): L40, M07.0–M07.3, or M09.0. Propensity score matching was conducted at a 1:5 ratio to enroll and define controls. The exclusion criteria were: a SARS-CoV-2 infection route originating from abroad, partial vaccination, or infection with SARS-CoV-2 within 14 days following full vaccination^10^. To mitigate the risk of identifying early pandemic cases, the NHIS only provided detailed information for cases occurring after 8 October, 2020. Thus, we excluded individuals diagnosed before this date.

### Operational definition of outcome

**Fig. 1:**
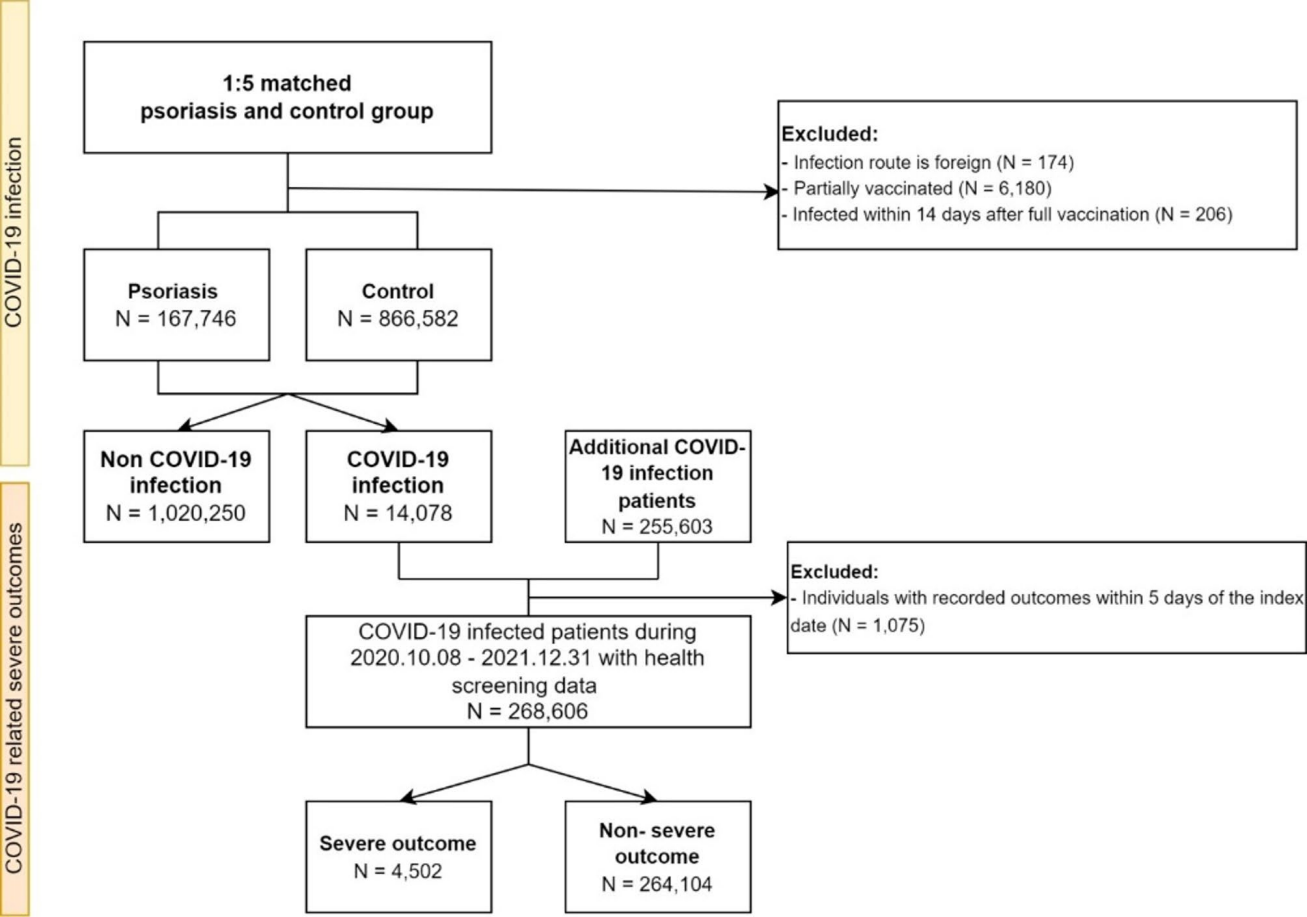
Flowchart illustrating the data collection process.

The primary outcome was the first SARS-CoV-2 infection. The secondary outcomes were severe complications associated with the infection, including death, application of invasive ventilation or oxygen therapy, and admission to the intensive care unit (ICU). The index date for the primary outcome was set as October 8, 2020, and the endpoint was determined as either the date of COVID-19 diagnosis or December 31, 2021. Regarding secondary outcomes, the starting date was the day of COVID-19 diagnosis; the endpoint was the day a severe outcome occurred, either within 60 days or 60 days after the starting date^11^. To mitigate potential bias resulting from individuals experiencing similar outcomes unrelated to COVID-19, we excluded patients with records of such outcomes within 5 days prior to their COVID-19 diagnosis^8^. To improve the statistical power, we included additional COVID-19 cases in the secondary analysis, utilizing the expanded dataset on COVID-19 cases. The detailed information is presented in Fig. 1.

### Operational definition of the treatment

Systemic treatment for psoriasis encompasses phototherapy, cyclosporine, methotrexate, acitretin, and biologics (including adalimumab, etanercept, guselkumab, infliximab, ixekizumab, secukinumab, and ustekinumab; Supplemental Table 1). Treatment thresholds (e.g., ≥ 4 prescriptions for methotrexate, ≥ 2 injections for biologics) were determined based on clinical prescribing patterns in Korea and intended to reflect sustained and therapeutically relevant exposure. Although specific cutoffs are not standardized across studies, our definitions align with those used in observational pharmacoepidemiologic research and are consistent with reimbursement and prescription frequency patterns in real-world clinical settings. Furthermore, we only included patients who sustained the same level of treatment throughout the follow-up period. Those who underwent multiple types of treatments, or who first started treatment after the index date, were excluded from the analysis. Patients who had never received any of the aforementioned systemic treatments were categorized into the nonsystemic group.

### Covariates

The covariates included age, sex, insurance level, residence (categorized as metropolitan area vs. other regions), body mass index, smoking status, Charlson comorbidity index, COVID-19 vaccination status (categorized as unvaccinated, fully vaccinated, or booster shot vaccinated), and comorbidities. Comorbidities included asthma, cardiovascular disease, chronic kidney disease, chronic obstructive pulmonary disease (COPD), cerebrovascular disease, diabetes mellitus, and hypertension. Throughout the study period, metropolitan areas experienced a substantial increase in COVID-19 cases, leading to the implementation of different policies — such as social distancing measures — compared with other regions. Therefore, we categorized residences into two groups: metropolitan areas and other regions. The vaccine immunity period was defined as 6 months after full or booster shot vaccinations^12^. In the secondary analysis, we incorporated additional information regarding the timing of the SARS-CoV-2 infection, considering that disease severity might be influenced by limited hospital resources during peak COVID-19 outbreaks. To address baseline imbalances and potential confounding, multivariable Cox regression models were adjusted for all listed covariates, including comorbidities. Furthermore, propensity score matching (1:5) was applied prior to analysis based on age and sex to reduce selection bias. Residual confounding due to imbalances in other characteristics was addressed through additional covariate adjustment in the multivariable models.

### Statistical analysis

We evaluated the baseline characteristics of all covariates using Pearson’s chi-squared test for categorical variables, and the Student’s t-test for continuous variables. Cox proportional hazard regression was utilized to compare clinical outcomes, with the vaccine immunity period considered a time-varying covariate. The proportional hazard assumption was verified to be met.

To assess differences in the incidence of COVID-19 during the vaccine immunity period among various subgroups, we conducted an interaction analysis using the extended self-controlled case series (SCCS) method. As a case-only design, the SCCS method enabled the estimation of the relative incidence (RI) between periods with and without a vaccine effect. Given the importance of reducing selection bias in studies where the vaccine is the main factor^13^, the SCCS method was the design of choice. Additionally, considering the observed tendency to avoid vaccination right after COVID-19, a 90-day pre-exposure washout period was implemented.

All statistical analyses were performed using R software (version 4.0; R project, Vienna, Austria), SAS Enterprise Guide (version 7.15; SAS Institute Inc., Cary, NC, USA), and Rex (version 3.5.3; RexSoft, Seoul, Republic of Korea)^14^. Statistical significance was set at *P* < 0.05.

### Ethical approval

This study was approved by the Institutional Review Board of the Konkuk University Medical Center (KUMC 2021-12-033), and a waiver of informed consent was granted as this study used de-identified data. All methods were performed in accordance with relevant guidelines and regulations, as outlined in the Declaration of Helsinki.

## Results

### Baseline characteristics

For the primary analysis, 167,746 patients with psoriasis were included and matched with 866,582 controls (Fig. 1). The COVID-19 incidence rate was 1.4% for both groups. The baseline characteristics of the cohort are presented in Table 1. The distributions of age (55.0 ± 15.1; *P* = 0.125) and sex (59.7% for male; *P* = 0.416) were not significantly different, as these were used as propensity score matching variables. However, patients with psoriasis exhibited a significantly higher prevalence of comorbidities for every disease under consideration (*P* < 0.001). For the secondary analysis, among those diagnosed with COVID-19, the study included 3,131 patients with psoriasis and 265,475 controls. Each summary of characteristics is presented in Supplemental Table 2.

**Table 1:** Demographic statistics of the study population.

	Control	Psoriasis	*P*-value
	(*N* = 866,582)	(*N* = 167,746)	
Age (years)	55.0 ± 15.1	55.1 ± 15.1	0.125
Sex			0.416
Male	517,168 (59.7%)	99,930 (59.6%)	
Female	349,414 (40.3%)	67,816 (40.4%)	
Insurance level	12.1 ± 6.0	11.3 ± 6.2	< 0.001
Residence			< 0.001
Metropolitan area	406,677 (46.9%)	80,386 (47.9%)	
Others	459,905 (53.1%)	87,360 (52.1%)	
Body mass index	24.4 ± 3.5	24.7 ± 3.6	< 0.001
Smoking			< 0.001
Never	515,733 (59.5%)	89,301 (53.2%)	
Former	179,575 (20.7%)	36,159 (21.6%)	
Current	171,274 (19.8%)	42,286 (25.2%)	
Comorbidity			
Asthma	160,689 (18.5%)	40,262 (24.0%)	< 0.001
Cardiovascular disease	78,810 (9.1%)	19,764 (11.8%)	< 0.001
Chronic kidney disease	13,888 (1.6%)	3761 (2.2%)	< 0.001
COPD	113,998 (13.2%)	28,811 (17.2%)	< 0.001
Cerebrovascular disease	38,925 (4.5%)	9,245 (5.5%)	< 0.001
Diabetes mellitus	211,500 (24.4%)	50,756 (30.3%)	< 0.001
Hypertension	301,107 (34.7%)	66,487 (39.6%)	< 0.001
Charlson comorbidity index			< 0.001
0	419,031 (48.4%)	66,290 (39.5%)	
1	194,985 (22.5%)	39,402 (23.5%)	
≥ 2	252,566 (29.1%)	62,054 (37.0%)	
Vaccine			< 0.001
Unvaccinated	55,017 (6.3%)	11,419 (6.8%)	
Full vaccinated	542,615 (62.6%)	103,374 (61.6%)	
Booster shot vaccinated	268,950 (31.0%)	52,953 (31.6%)	
Mean follow-up (days)	447.4 ± 19.6	447.4 ± 19.5	
COVID-19 infection	11,770 (1.4%)	2308 (1.4%)	

COPD, chronic obstructive pulmonary disease.

### Survival results for clinical outcomes

**Fig. 2:**
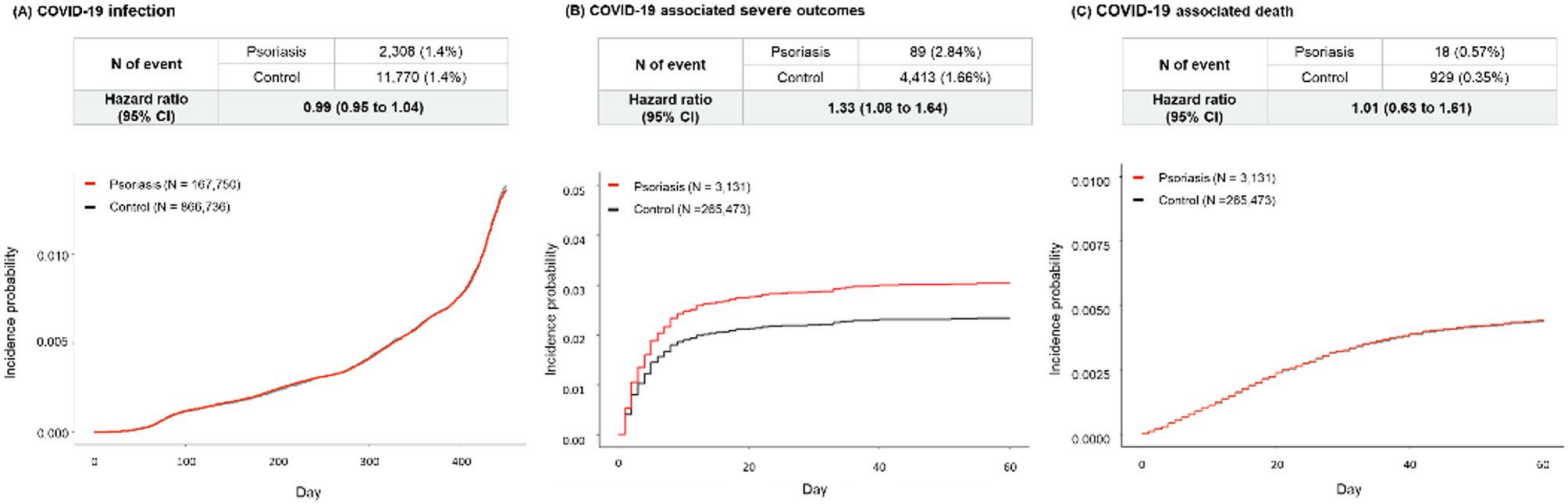
Survival results for COVID-19 and associated severe outcomes.

In the primary analysis, we found no association between psoriasis and COVID-19 (adjusted hazard ratio [aHR]: 0.99, 95% confidence interval [CI]: 0.95–1.04; Fig. 2A). We identified patients with COVID-19 who were immediately hospitalized following diagnosis and conducted additional analyses considering only these cases as the outcomes. This yielded similar results, with no significant association observed (aHR: 0.97; 95% CI: 0.90–1.04; Supplemental Fig. 1). Conversely, in the secondary analysis, patients with psoriasis exhibited significantly higher incidence rates for severe outcomes associated with COVID-19 (aHR: 1.33, 95% CI: 1.08–1.64; Fig. 2B). However, this relationship was not significant when only death was considered as the outcome (aHR: 1.01, 95% CI: 0.63–1.61; Fig. 2C).

**Fig. 3:**
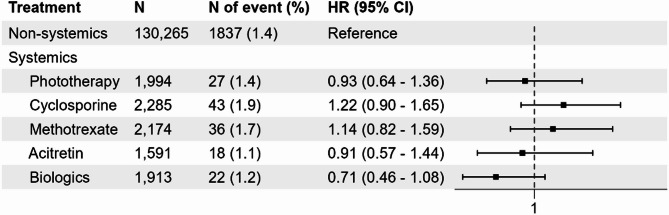
Survival results for COVID-19 in patients with psoriasis categorized by treatment.

We categorized patients with psoriasis according to their treatments. Subgroup analyses were then performed to identify any groups susceptible to the infection (Fig. 3). Compared with the nonsystemic group, the cyclosporine (aHR: 1.22, 95% CI: 0.90–1.65) and methotrexate (aHR: 1.15, 95% CI: 0.82–1.59) groups showed a higher incidence of COVID-19. Conversely, the phototherapy (aHR: 0.93, 95% CI: 0.64–1.63), acitretin (aHR: 0.91, 95% CI: 0.57–1.44), and biologics (aHR: 0.71, 95% CI: 0.46–1.08) groups exhibited a lower incidence of COVID-19. However, these differences were not statistically significant. In summary, no significant increase was observed regarding the incidence of COVID-19 among the various treatment groups compared to the nonsystemic group.

### Interaction analysis of the vaccine effect

We investigated whether the RI of COVID-19 was significantly lower during the vaccine immunity than control period (Table 2). The results demonstrate that the vaccine had a significant protective effect (RI: 0.43, 95% CI: 0.40–0.46). Subsequently, we assessed the interaction effects between the vaccine immunity period and each treatment (*P* > 0.05 for all) or psoriasis (*P* = 0.25). These interactions were not statistically significant, suggesting no notable difference in the protective effect of the vaccine between the psoriasis and control groups, as well as between the systemic and nonsystemic groups.

**Table 2:** Interaction effect estimates between the vaccine immunity period and psoriasis or treatments.

	Vaccine effect	
	Relative incidence	P-value
Overall	0.43 (0.40 to 0.46)	< 0.001
Within psoriasis	0.46 (0.37 to 0.56)	< 0.001

	Interaction effect	
	Effect size	*P*-value
Overall		
Control (ref)	0	
Psoriasis	0.08 (− 0.05 to 0.21)	0.25
Within psoriasis		
Non-systemics (ref)	0	
Systemics		
Phototherapy	− 0.62 (− 1.73 to 1.74)	0.28
Cyclosporine	− 0.29 (− 1.13 to 0.55)	0.5
Methotrexate	− 0.98 (− 2.04 to 0.08)	0.07
Acitretin	0.36 (− 0.99 to 1.71)	0.6
Biologics	− 0.56 (− 1.81 to 0.69)	0.38

## Discussion

Psoriasis can increase susceptibility to infection due to immunological abnormalities and the use of immunosuppressants^2^. However, the correlation between psoriasis and COVID-19 remains unclear due to conflicting findings. Consistent with our findings, Cho et al.^15^ documented that patients with psoriasis were not significantly more likely to contract COVID-19 than the general population. A review revealed no clear link between IMIDs, including psoriasis, and COVID-19^7^. Nonetheless, a nationwide US study demonstrated an elevated risk of COVID-19 among patients with psoriasis (adjusted odd ratio [aOR]: 1.18, 95% CI: 1.13–1.23)^16^. Another US study highlighted an increased susceptibility to COVID-19 in patients with psoriasis (OR: 1.48, 95% CI: 1.06–2.07)^17^. These findings could potentially mirror disparities in quarantine policies between countries, including measures like social distancing. Furthermore, the inclination of individuals with diseases to adopt more rigorous protective measures might also impact their vulnerability to infection^18^.

This study found a heightened risk of severe COVID-19 outcomes — including ICU admission, invasive mechanical ventilation, and death — in patients with psoriasis. Comorbidities, including psoriasis, have been linked to an increased risk of severe COVID-19^19^. A UK cohort study revealed that patients with IMIDs, including psoriasis, had an increased likelihood of adverse COVID-19 outcomes^8^. The risk of mortality was highest among individuals with inflammatory joint disease. A Danish population-based study revealed an elevated risk of COVID-19-related hospitalization and mortality among patients with IMIDs: one in three were hospitalized, and one in ten died due to COVID-19^9^. In a Korean national cohort study, the risk of severe COVID-19 was increased in patients with autoimmune rheumatic diseases, including psoriatic arthritis^20^. A multinational registry-based cohort study identified older age, male sex, nonwhite ethnicity, and chronic lung disease as risk factors for severe COVID-19 in patients with psoriasis, similar to the general population^21^. Complex biopathological mechanisms resulting from multiple cytokines and immunological abnormalities may lead to these devastating sequelae caused by SARS-CoV-2 infection in patients with autoimmune diseases^20^.

These findings imply that individuals with psoriasis may be more susceptible to severe COVID-19. Managing the risk of COVID-19 among patients with psoriasis requires individualized treatment strategies, accounting for age, sex, and comorbidities. This perspective could contribute to establishing guidelines for the response to COVID-19 in patients with autoimmune diseases, including psoriasis.

Immunomodulators are widely used for the treatment of psoriasis and may lead to reduced resistance to infections among these patients^6,22,23^. French and Italian cohort studies suggested increased COVID-19-related hospitalization rates in patients with psoriasis undergoing systemic therapy^24,25^. However, numerous studies investigating the link between immunomodulatory agents, including biologics, and COVID-19 or its severity in patients with psoriasis have found no significant associations^15,26–28^. Similarly, the current study found no link between medication use and COVID-19 susceptibility in patients with psoriasis. A large-scale cohort study of IMIDs also demonstrated no correlation between the use of targeted immune-modifying drugs and the severity of COVID-19^8^. Some reports indicate that immunomodulatory therapy could mitigate post-COVID-19 complications, such as cytokine storms^29^. A Danish study identified a diminished risk of severe COVID-19 among patients with IMIDs receiving biologics^9^. In particular, TNF inhibitors demonstrated a reduced risk of hospitalization in patients with psoriasis. This protective effect may be attributed to their potential to inhibit TNF-ɑ, which plays a critical role in severe COVID-19^21,30,31^. These findings support guidelines recommending ongoing immunomodulatory therapy for dermatological patients during the COVID-19 pandemic.

This study revealed that the effectiveness of COVID-19 vaccines in patients with psoriasis, as evidenced by confirmed infections, was comparable to that in the control group. Importantly, the use of immunomodulators in patients with psoriasis did not significantly impact the effectiveness of COVID-19 vaccines. Studies on the immunogenicity of COVID-19 vaccines in patients with psoriasis showed no correlation between methotrexate/biologic use and antibody titers^32,33^. However, certain studies noted reduced vaccine-specific immune responses in patients with psoriasis treated with TNF inhibitors^34,35^. Other studies found that patients with IMIDs treated with methotrexate exhibited diminished antibody responses to vaccines^36–38^.  A randomized controlled trial demonstrated enhanced antibody responses to vaccines in patients with IMIDs after discontinuing methotrexate for 2 weeks^39^. These findings suggest that the immunosuppressive effects of psoriasis treatments may reduce vaccine efficacy. Further research is necessary to validate the clinical significance of antibody levels and their role in COVID-19 susceptibility, considering the potential effects of immunomodulators on vaccine efficacy.

This study has several strengths. First, it was a large-scale population-based study analyzing the causal relationship between psoriasis and COVID-19, encompassing the entire psoriasis population, irrespective of treatment; all definitions of COVID-19 cases were based on official data provided by Korean government agencies. Second, our dataset includes comprehensive information on patients with COVID-19 — including the route of transmission, date of confirmation, date of death, reporting region, and vaccine details — allowing for thorough analysis encompassing these critical variables. Third, the high vaccination rates (> 90%) within our study population provided a sufficient sample size for performing vaccine-related interaction analyses.

However, certain limitations need to be considered. Due to the nature of claims data, disease determination relies on diagnostic codes, potentially resulting in misclassification. The integration of KDCA data in this study helped mitigate misclassification risks and enhance the overall accuracy of the study results. Additionally, clinical characteristics — such as disease severity and laboratory test results — are not available in the NHIS database. Moreover, the possibility of residual confounding, particularly confounding by indication, should be considered. Patients receiving systemic therapies, such as methotrexate or biologics, may have more severe psoriasis or additional unmeasured clinical complexity. Although we adjusted for a wide range of demographic and comorbidity variables, the lack of direct measures of disease severity in the claims database limits the ability to fully account for this bias. Additionally, demographic differences in the prevalence of psoriasis (Korea, 0.44–0.45%^40^; Western countries, 2.0–4.0%^41^) could limit broader racial generalization.

In this nationwide cohort analysis, no significant differences in the risk of COVID-19 were observed between the psoriasis and control groups; however, patients with psoriasis had a higher risk of severe COVID-19. Additionally, no difference was observed regarding the risk of COVID-19 or the effectiveness of vaccines across various systemic immunomodulatory treatments among patients with psoriasis. Considering the association between psoriasis and the severity of COVID-19, dermatologists should be attuned to appropriate management strategies and responses both before and after COVID-19 infection.

## Electronic supplementary material

Below is the link to the electronic supplementary material.

Supplementary Material 1

Supplementary Material 2

Supplementary Material 3

## Data Availability

The datasets used and/or analyzed during the current study are not available from the corresponding author, because the dataset was obtained from the National Health Insurance Service, Republic of Korea.
