# Supplementary material for: COVID-19 susceptibility, severity, and vaccine effectiveness in patients with psoriasis: a nationwide cohort study in South Korea
Source: Sci Rep. 2025 Jul 15;15:25608. doi: 10.1038/s41598-025-06495-8 (PMC12263980; doi:10.1038/s41598-025-06495-8)
Supplement: Supplementary file 2 — Supplementary Material 2 [file 41598_2025_6495_MOESM2_ESM.docx]

**Table S1. Criteria for classifying each treatment 6 months prior to the index date**

| **Treatment** | **Criteria** |
| --- | --- |
| Phototherapy | At least 12 times |
| Cyclosporine | At least 28 days of prescription |
| Methotrexate | At least 4 times of prescription |
| Acitretin | At least 28 days of prescription |
| Biologics | At least 2 injections |

**Table S2. Baseline characteristics of individuals who experienced COVID-19 infection**

|  | Control | Psoriasis | P-value |
| --- | --- | --- | --- |
|  | (N=265,475) | (N=3,131) |  |
| **Age (years)** | 51.5 ± 15.0 | 55.2 ± 14.8 | <0.001 |
| **Sex** |  |  | <0.001 |
| Male | 131,762 (49.6%) | 1,741 (55.6%) |  |
| Female | 133,713 (50.4%) | 1,390 (44.4%) |  |
| **Insurance level** | 11.2 ± 6.0 | 11.3 ± 6.3 | 0.546 |
| **Residence** |  |  | 0.265 |
| Metropolitan area | 192,525 (72.5%) | 2,230 (71.2%) |  |
| Others | 72,950 (27.5%) | 901 (28.8%) |  |
| **Body mass index** | 24.5 ± 3.7 | 24.7 ± 3.5 | <0.001 |
| **Smoking** |  |  | <0.001 |
| Never | 177,503 (66.9%) | 1,882 (60.1%) |  |
| Former | 48,415 (18.2%) | 716 (22.9%) |  |
| Current | 39,557 (14.9%) | 533 (17.0%) |  |
| **Comorbidity** |  |  |  |
| Asthma | 17,150 (6.5%) | 347 (11.1%) | <0.001 |
| Cardiovascular disease | 15,283 (5.8%) | 292 (9.3%) | <0.001 |
| Chronic kidney disease | 2,856 (1.1%) | 64 (2.0%) | <0.001 |
| COPD | 13,128 (4.9%) | 253 (8.1%) | <0.001 |
| Cerebrovascular disease | 6,826 (2.6%) | 150 (4.8%) | <0.001 |
| Diabetes mellitus | 50,382 (19.0%) | 851 (27.2%) | <0.001 |
| Hypertension | 74,842 (28.2%) | 1,179 (37.7%) | <0.001 |
| **Charlson comorbidity index** |  |  | <0.001 |
| 0 | 133,070 (50.1%) | 1,183 (37.8%) |  |
| 1 | 58,380 (22.0%) | 667 (21.3%) |  |
| ≥2 | 74,025 (27.9%) | 1,281 (40.9%) |  |
| **Timing of the COVID-19 infection** |  |  | 0.014 |
| Non-peak | 33,064 (12.5%) | 361 (11.5%) |  |
| peak | 232,411 (87.5%) | 2,770 (88.5%) |  |
| **Vaccine** |  |  | <0.001 |
| Unvaccinated | 119,883 (45.2%) | 1,255 (40.1%) |  |
| Full vaccinated | 127,611 (48.1%) | 1,613 (51.5%) |  |
| Booster shot vaccinated | 17,981 (6.8%) | 263 (8.4%) |  |
| **Mean follow-up (days)** | 43.4 ± 20.8 | 41.9 ± 21.2 | <0.001 |
| **Occurrence of the severe outcome** | 4,413 (1.7%) | 89 (2.8%) | <0.001 |
