# Supplementary material for: COVID-19 susceptibility, severity, and vaccine effectiveness in patients with psoriasis: a nationwide cohort study in South Korea
Source: Sci Rep. 2025 Jul 15;15:25608. doi: 10.1038/s41598-025-06495-8 (PMC12263980; doi:10.1038/s41598-025-06495-8)
Supplement: Supplementary file 3 — Supplementary Material 3 [file 41598_2025_6495_MOESM3_ESM.docx]

**Figure legends:**

Supplementary Figure 1. Survival results for COVID-19 associated admission in patients with psoriasis
